# The Role of Hospital Transfer in Reexamination Computed Tomography Scans: A Nationwide Cohort Study of Gastric Cancer Patients Undergoing Surgery

**DOI:** 10.3390/healthcare8010002

**Published:** 2019-12-19

**Authors:** Jaeyong Shin, Yoon Jung Choi, Young Choi, Sang Gyu Lee, Ji Man Kim

**Affiliations:** 1 Department of Preventive Medicine, Ajou University School of Medicine, 164 Worldcup-ro, Yeongtong-gu, Gyeonggi-do 16499, Korea; drshin@ajou.ac.kr (J.S.); choiyoung@yuhs.ac (Y.C.); 2 Health Insurance Review and Assessment Service, 60 Hyeoksin-ro, Wonju, Gangwon-do 26465, Korea; yneschoi@hira.or.kr; 3 Department of Preventive Medicine, Yonsei University College of Medicine, 50-1 Yonsei-ro, Seodaemun-gu, Seoul 03722, Korea; leevan@yuhs.ac; 4 Graduate School of Public Health, Yonsei University, 50-1 Yonsei-ro, Seodaemun-gu, Seoul 03722, Korea

**Keywords:** computed tomography, high-tech diagnostic utilization, reexamination

## Abstract

Because the high-cost of medical imaging can cause a tremendous economic burden across the health care system, we investigated factors associated with taking additional computed tomography (CT) scans. Data of gastric cancer patients were eligible for analysis if the patient underwent a gastrectomy during the study period (2002–2013). We defined initial CT scans as those taken within 90 days from the surgery date. If there was an additional CT scan between the date of an initial CT scan and the surgery date, we regarded it as a reexamination. We used multivariate logistic regression analysis for reexamination CT scans. Among 3342 gastrectomy patients, 1165 participants underwent second CT scans. Transfer experience (adjusted odds ratio (OR) = 23.87, 95% confidence interval (CI) = 18.15–31.39) was associated with higher OR for reexamination. Among transferred patients, an increased number per 100 beds at the initial CT hospital was associated with a decreased OR for reexamination (OR = 0.88, 95% CI = 0.83–0.94), but increased beds in surgery hospitals was related to an increased OR for reexamination (OR = 1.29, 95% CI = 1.20–1.36). In our study, transfer experience, initial CT scan in a low-volume hospital, and surgical treatment in a high-volume hospital were associated with reexamination CT scans.

## 1. Introduction

Computed tomography (CT) is a widely used medical imaging technique in Korea [1,2]. According to OECD (Organisation for Economic Co-operation and Development) health data, 7,298,133 CT exams were performed in Korea in 2013 [3]. In addition, 37.6 CTs per million population were performed in Korea in 2013—the fourth highest among OECD countries [3]. As CT scans have improved gradually, they have become more important in detecting malignant lesions in internal organs [4].

The first commercially viable CT scanner was invented by Sir Godfrey Hounsfield in Hayes, United Kingdom, at EMI (Electric and Musical Industries) Central Research Laboratories using X-rays. Hounsfield conceived his idea in 1967 [5]. In Korea, the first CT scanners were available for clinical practices in the late 1970s and early 1980s [6]. After their first adoption, more advanced CTs were developed, and the quality and resolution of images improved dramatically [7]. Therefore, older and lower quality CT scanners might be causing a need for reexamination CTs. The proportion of CT scanners over 10 years old is 23.6%, and 34.2% are reused CT scanners [8].

The Korean Ministry of Health and Welfare (MoHW) regularly tests the quality of CT scanners based on enforcement regulations of the Medical Service Act. Although the currently used CT scanners satisfy the minimal quality standards, there are still some issues in quality across CT scans. Moreover, the old and reused CT scanners are usually owned by small-volume hospitals in rural areas [9]. Thus, we sought to investigate whether these old and reused CT scanners were truly associated with reexamination CT scans. Moreover, we examined what other factors were related to repeated scans.

To assess this, we selected patients with gastric cancers who underwent a gastrectomy, with or without chemotherapy. We targeted this population because these patients were eligible to undergo CT scans before their operations [10,11], and gastric cancer is common in Korea [12,13].

## 2. Materials and Methods

### 2.1. Data Source and Collection

All citizens in Korea are obligated to enroll in a single-payer, national health insurance and medical aid program administered by the National Health Insurance Corporation (NHI) [14]. The NHI database includes information regarding basic patient demographics, reimbursement for medical services, disease codes according to the International Classification of Diseases (ICD-10), identifiers for the clinic or hospital, medical history, and mortality. These data are then automatically linked to the Korean National Statistical Office, where they are obtainable for research purposes. The present study used data from the National Health Insurance Service-National Sample Cohort (NHIS-NSC) [15], which included 1,025,340 representative subjects (~2.2% of the country’s population), who were randomly stratified and selected based on age, gender, insurance type, income, residential region, and individual total medical costs.

This study was exempt from institutional review board evaluation. However, the study protocols adhered to the guidelines outlined in the Declaration of Helsinki.

### 2.2. Description of the Study Cohort

**Figure 1:**
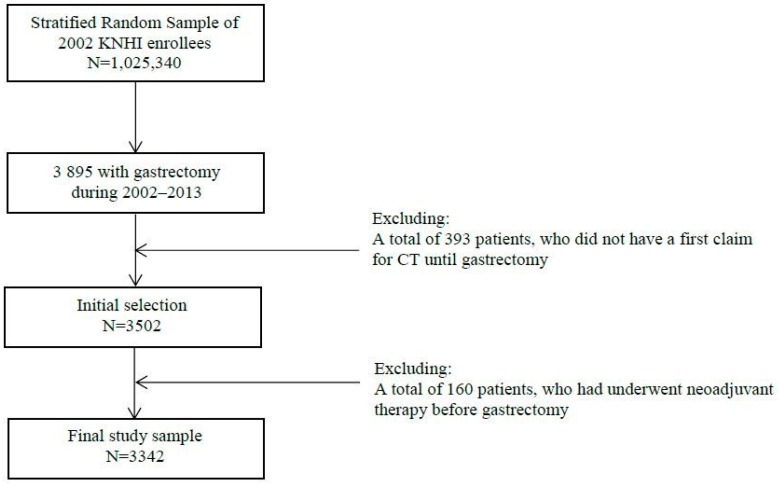
Participant selection.

Patients’ data were eligible for analysis if the patient was diagnosed with gastric cancer (ICD-10 code, C16), underwent a gastrectomy during the study period (2002−2013), and was >20 years old at the time of diagnosis. We excluded patients who had another primary cancer or known metastatic disease at the time of diagnosis. A schematic flow chart for patient selection is shown in Figure 1. In total, 3895 patients met the criteria. However, because 393 patients had no history of CT scans, the initial population for the study was 3502 patients. We also excluded a further 160 patients who had undergone neoadjuvant chemotherapy before surgical resection. Finally, a total number of 3342 patients were enrolled in this study.

### 2.3. Initial and Reexamination CT Scans

**Figure 2:**
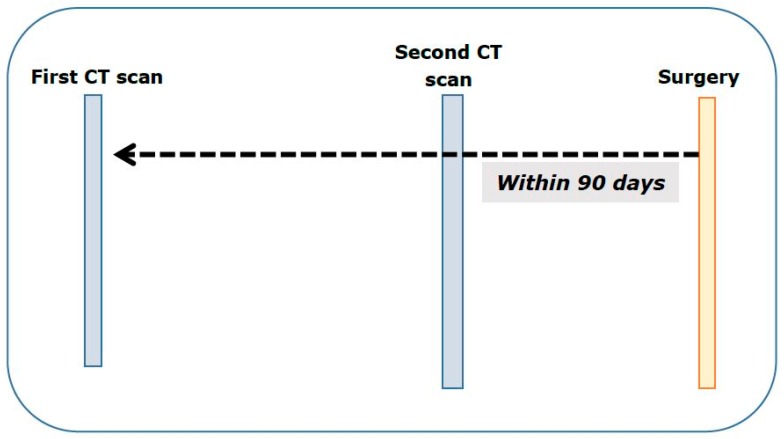
Initial and reexamination computed tomography (CT) scans.

All included CT scans were at abdominal–pelvic level according to the Electronic Data Interchange code for claims to the national health insurance scheme. We defined initial CT scans as those taken before 90 days from the surgery date. Although operable gastric cancer is not necessary for acute management, in general, we assumed that most gastric cancer patients would proceed to a gastrectomy as quickly as possible, within 90 days. If there was any abdominal–pelvic CT scan claim data between the date of the initial CT scan and the surgery date, we regarded that procedure as a reexamination CT scan (Figure 2).

### 2.4. Statistical Analysis

Demographic characteristics, including age, gender, health coverage, premium level, residential area, surgical type (total/subtotal), and transfer history for surgery, were included in the data. The NHI premium was used as a proxy measure of income because it is proportional to monthly income, including earnings and capital gains. The income deciles of enrolled subjects were categorized into the following three groups: ‘Low’ for deciles 0–3, ‘Middle’ for deciles 4–7, and ‘High’ for the deciles 8–10. Some patients might have needed to undergo CT scans two or more times due to other medical conditions. Thus, we determined the Charlson comorbidity index (CCI) for each patient, a widely used indicator of fatal medical conditions [16,17]. It predicts the one-year mortality for a patient who may have a range of comorbid conditions, such as heart disease, AIDS (Acquired Immunodeficiency Syndrome), or cancer (a total of 22 conditions). Each condition is assigned a score of 1,2,3, or 6, depending on the risk of dying associated with each one. Scores are summed to provide a total score to predict mortality.

We also considered the level of hospitals where patients received the initial and reexamination CT scans and categorized them into three levels: general hospitals, hospitals, and clinics. Theoretically, Korea has a well-organized delivery system, with three levels of hospitals including, clinic, hospital, and general hospital. According to their definition in related laws, a clinic means a health care organization in which medical professionals can perform their medical services under certain circumstances. It is similar to a private clinic in other countries. In terms of a hospital, it is defined as health care facilities with over 30 beds and providing medical services from clinical specialists. It usually covers 5000 to 50,000 people. In Korea, a general hospital is a highly professional health care organization with over 200 beds, covering 50,000 to 500,000 people. There are a lot of sub-specialty clinicians and they usually have a responsibility for rare and intractable diseases. The locations of the hospitals and their numbers of beds were included as indicators of medical performance.

Descriptive statistics were computed for all variables as frequencies and percentages for categorical variables using a χ^2^ test. To investigate the association between variables of interest and reexamination CT scans, we used a multivariate logistic regression analysis, with full adjustment for possible demographic and hospital variables, as above. We also performed a sensitivity analysis using specific transfer routes. Odds ratios (ORs) and 95% confidence intervals (95% CIs) were calculated, and statistical significance was set at *p* < 0.05. We conducted all statistical analyses using the SAS software (version 9.3; SAS Institute Inc., Cary, NC, USA). All the variables were adjusted for the statistical analyses.

## 3. Results

### 3.1. Demographic Characteristics

Among 3342 operable gastric cancer patients, 1165 participants underwent second CT exams before surgery from April 2002 to December 2013 (Table 1). As the age groups became older, the probability of reexamination CT scans increased. In terms of severity in general health conditions, patients with reexamination CT scans had higher CCI scores.

In total, 679 patients (20.3%) were transferred to a different hospital between the diagnosis and surgical treatment for gastric cancer. When patients were transferred, reexamination CT scans were more frequent. In terms of hospital characteristics where the initial CT scans were taken, general hospitals and locations within the capital city, Seoul, had lower probabilities of reexamination scans.

### 3.2. Multivariate Logistic Regression for Reexamination CT Scans

To measure the adjusted odds ratios (ORs) for reexamination CT scans, we used multivariate logistic regression (Table 2). According to the results, transfer experience (OR = 23.87, 95% CI = 18.15–31.39) and total gastrectomy (OR = 1.90, 95% CI = 1.55–2.33) were associated with higher adjusted odds ratios for reexamination scans compared with the respective reference group. In contrast, undergoing the initial CT at a clinic (OR = 0.45, 95% CI = 0.26–0.80) was related to a lower odds ratio compared with at general hospitals.

### 3.3. Subgroup Analysis for Transferred Patients Only

Because the odds ratio was highest for transferred patients, we examined them separately. To determine the difference in transfer routes, patients who transferred (1) from a clinic or a hospital to a general hospital or (2) from a general hospital to another general hospital were included in this subgroup analysis. Only eight patients were excluded via this process, and 671 patients were included in this subgroup analysis. Moreover, we investigated some additional variables of interest, such as the number of beds in the hospital of the initial diagnosis and in the hospital where the surgery occurred.

According to this sensitivity analysis (Table 3), no statistically significant difference was found in transfer route as assessed by the adjusted odds ratio for reexamination scans. However, the numbers of beds in the initial CT hospital and in the surgical treatment hospital were both related to repeated CT scans. Regarding the initial CT hospital, an increased number of beds was associated with a decreased odds ratio for reexamination (OR = 0.88, 95% CI = 0.83–0.94, per 100 beds). In contrast, an increased number of beds in the surgical treatment hospital was related to an increased odds ratio for reexamination (OR = 1.29, 95% CI = 1.20–1.36, per 100 beds).

### 3.4. Subgroup Analysis: Transfer between General Hospitals

We also investigated whether the number of beds was still associated with reexamination CT scans when the initial CT hospital was at the general hospital level. Because general hospitals need accreditation from the government, including medical device examinations, we needed to investigate transferred patients at the same level of hospital. According to the results (Table 4), the odds ratio for number of beds at the initial CT hospital (OR = 0.91, 95% CI = 0.88–0.95, per 100 beds) and at the surgical treatment hospital (OR = 1.10, 95% CI = 1.06–1.13, per 100 beds) still showed statistically significant differences.

**Figure 3:**
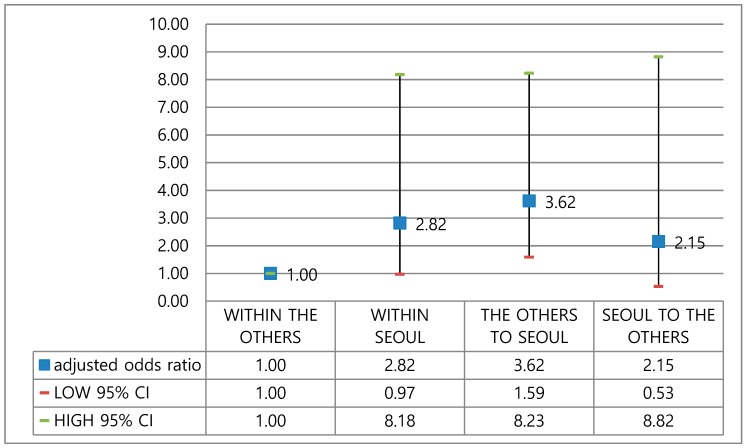
Adjusted odds ratios for reexamination CT scans for patients experiencing transfer between general hospitals.

We also examined geographical transfers between Seoul and other provinces, including transfers (1) between other provinces; (2) within Seoul; (3) from the other provinces to Seoul; and (4) from Seoul to other provinces (Figure 3). Because the four largest and best-known hospitals are located in Seoul, many cancer patients prefer to undergo surgery in Seoul. Patients transferred from outside Seoul to Seoul (OR = 3.62, 95% CI = 1.59–8.23) showed the highest odds ratio for reexamination CT scans.

## 4. Discussion

A CT scan is one of the best ways to make an operation plan for gastric cancer patients [18,19,20]. Although endoscopic ultrasound and laparoscopy are invasive testing procedures, they are effective in assessing the range of gastric wall invasion by gastric cancer [21]. Further, they visualize invasion of gastrointestinal tissues or adjacent organs and swollen lymph nodes around the gastrointestinal tract. However, unlike endoscopic ultrasound and laparoscopy, CT scan is an invasive procedure that can generate an objective image of the entire abdomen including the gastrointestinal and adjacent organs, based on local staging and remote metastasis [22]. According to the data, almost all of the gastric patients undergoing surgery had a CT scan, and 35.3% had two or more scans before surgery. In the multivariate analysis, a hospital transfer was highly associated with reexamination CT scans. Among the transferred patients, small initial hospitals and high-volume surgery hospitals were related to higher probabilities of reexamination CT scans. When we only analyzed transfers within general hospitals, where we hypothesized that the initial and final CT scans might be equal, only transfers from other provinces to Seoul, the Korean capital, showed a statistically significant increased odds ratio.

We could not explain why 35% of patients underwent reexamination CT scans. However, some possible hypotheses can be suggested. First, the quality of initial CT scans might not have been appropriate for making a surgical plan. Low imaging quality in CT scans may hinder the surgeon from establishing the staging and surgery method. According to national published data [9], 37.7% of CT scanners in primary clinics and 24.6% in hospitals were used less than once per day, whereas this was true for only 0.9% of scanners in general hospitals. With such low use rates of use, it is not easy to maintain consistent CT quality or to justify the acquisition of a more advanced scanner. Thus, it might be carefully assumed that the low use rates for CT scanners might be associated with low-quality CT imaging, resulting in the need for additional CT scans. However, in our study, the patients who had taken the initial CT scan at primary clinics had lower association to re-examination of CT scan. Although the number of these patients are very small (2.3%), it seems reasonable to consider other plausible explanations for the relationship.

Second, a hospital might avoid performing the initial CT for several financial reasons. Under the fee-for-services system, hospitals have typically tended to increase their medical activities, including CT examinations [23]. This has also been observed in other countries. According to a study from the US [24], stable coronary artery disease (CAD) patients, who were diagnosed with new coronary CT angiography, were more likely to undergo subsequent invasive procedures and have higher CAD-related spending than others who underwent stress testing, regardless of disease severity. The authors mentioned that the additional imaging resulted in not only the added direct costs of the CT scan but also the indirect costs for subsequent medical procedures. They named this phenomenon a “diagnostic-driven treatment cascade.”

Third, some patients may bring their own medical images, which may lead to mistakes although the chance is very low. Under the health care system in Korea, all patients are expected to bring their medical certificates for referrals to tertiary hospitals from their doctors at private clinics or other hospitals. In addition, they are expected to ask for copies of medical images from the medical staff and bring them other facilities. While this process may seem to be easy for many people, it may be a complicated step for others, such as those with disabilities and elderly patients.

Within the Korean national health care system, the ‘Health Insurance Review and Assessment Service’ (HIRA) monitors medical claims and conducts quality assessments for medical fee verification [25]. In 2003, the agency suggested and permitted a ‘reading fee for external medical images’ to prevent unnecessary CT scans and increase efficiency in medical resource use [26]. This insurance fee allowed 20% of the original CT costs for reading and interpretation in another hospital by a radiologist [26]. Through this, the agency hoped to encourage behavior changes in hospitals to avoid their performing another CT scan for profit, regardless of the patient’s medical condition and the imaging quality of the initial CT scan.

However, contrary to the agency’s expectations, it seems that there is still considerable inefficiency in the use of high-cost medical imaging devices. In Korea, there is still insufficient insurance coverage for magnetic resonance imaging (MRI) or positron emission computed tomography (PET). Thus, the government agency is not able to review reexamination rates for MRI and PET images [27]. Even with CT scans, which are mostly covered by the national health insurance scheme, over 20% of scans were reexamination scans in 2010. Although more focus interviews and other quantitative analyses are needed to explain the potentially wasteful use of medical resources, all three hypotheses mentioned above are likely associated with the outcome.

To overcome and improve the current situation, we suggest several solutions. Through policy synchronization, the quality problem can be resolved when the pursuit of micro-efficiencies by providers are synchronized with the macro-efficiency needs of the national health system. If the government discloses quality information for high-tech devices and gives incentives for provider efforts to increase quality, the current competition between providers to capture patients could evolve into a competition for better quality. In addition to this vertical synchronization, horizontal policy synchronization, such as with NHI policy, should also be discussed. 

For example, in the US, the Center for Medicare and Medicaid Services (CMS) decided that all CT and MRI images should be performed using accredited medical devices based on the Medicare Improvements for Patients and Providers Act of 2008 (MIPPA) [28]. According to the Act, three independent organizations—the American College of Radiology, the Intersocietal Accreditation Commission (IAC), and the Joint Commission (JC)—have the authority to certify the quality of imaging devices. In addition, the Australian Medicare system requires two accreditations from the Diagnostic Imaging Accreditation Scheme and a location-specific practice number to claim for medical images [29].

We also suggest that a long-term strategy for balancing CT scan supply and demand should run parallel with a quality management strategy. As we already mentioned, the inefficient usage of CTs in primary clinics and hospitals may be related to difficulty in quality maintenance. Since the Korean health care system is mainly operated by private health care providers and hospitals, it is not easy to allocate medical resources efficiently. However, the government has to prepare long-term strategies to re-allocate high-cost medical devices via elaborating health care delivery systems. 

Advanced internet technologies (IT) and information and communications technologies (ICT) generally could provide an alternative. That is, high-tech medical images could be made anywhere and could be shared via digital storage. Then, they could be readily transmitted, and patients would not need to copy and bring them to another hospital. Moreover, all images could be evaluated easily, and real-time feedback on quality could be provided via the system. However, the ‘Personal Information Protection Act’ does not allow the sharing of personal medical records without permission [30], and there is still a risk of someone hacking into the network. Despite these limitations and concerns and the need to use ICT carefully, it could dramatically increase efficiency in the health care system overall [31]. To encourage this, the vertical and horizontal synchronization of health policies should support greater adoption of ICT in health care [6].

This study has several limitations. First, we did not consider use of contrast agents. Usually, approximately 40% of all CT scans use a contrast agent for better delineation of soft tissue contrast. Contrast agents are widely used in CT and MRI, as they enhance the contrast of tissues and improve diagnostic accuracy. For this reason, contrast agents are the most frequently used agents among intravenous agents [32]. Second, we did not determine any concrete reason for reexamination CT scans. Only claim data were used in the analysis; further qualitative investigations are needed. Third, caution is needed before generalizing these results to other diseases. Although we targeted the second most common cancer in Korea, the pattern of care in other diseases could differ. Forth, we did not adjust for medical conditions, including cancer stage. If the stage is more advanced, there may a higher probability of further CT scans. We tried to overcome this problem by adjusting the surgical type, such as total versus subtotal/partial gastrectomy. However, since a surgical procedure is dependent on tumor location basically, we admit that this is still not enough to reflect the disease severity exactly.

Despite these limitations, our study has several strengths. First, we used national cohort data from a random sample. Thus, the result may have high external validity in Korea. Second, gastric cancer is the second most common malignant neoplasm in Korea. Thus, the patterns of care in other severe cancers, which also need high-tech medical images, would be expected to be similar. Third, to our knowledge, there are few academic reports on reexamination CT scans in Asian countries. The growth rate in the health care systems is increasing in keeping with increased incomes and growing medical needs in this region; we have made suggestions as to how to provide and control high-quality medical images efficiently.

Conventional CT, helical CT, and fusion imaging technology such as Positron Emission Tomography (PET)-CT are currently used to diagnose and treat gastric cancer. Further, various imaging devices that can detect diverse biological information are utilized in clinical practice. Such equipment can measure gastric cancer-related information that could not be measured with existing equipment therefore they are used to diagnose gastric cancer and to determine therapeutic effects and prognostic prediction [33]. Computed tomography scans with advanced technology would boost the efficiency and quality of diagnosis and treatment of gastric cancer. Reexamination CT scans may be needed to examine changes in the state and to devise treatment plans for patients. When considering reexaminations, imaging technologies that can detect diverse biological information, such as MRI and PET-CT, are also used in clinical practice. Therefore, continuous attention to novel technology as well as a comprehensive understanding and approach will be needed in molecular biology and tumor biology.

## 5. Conclusions

Although the CT scanner is a widely used medical imaging device, associated quality measures have not been performed well. In our study, patient transfers, low-volume hospitals for the initial CT scan, and high-volume hospitals for surgical treatment were associated with reexamination CT scans. Because the high cost of medical imaging can cause a tremendous economic burden on the entire health care system, there is a need to increase the quality of CT scans using effective political and ICT methods.

## Figures and Tables

**Table 1:** The demographic characteristics for the enrolled gastrectomy patients from April 2002 to December 2013.

Variable	Reexamination CT Scan				Total	*p*-Value
	NO		YES			
	N	%	N	%		
Age Group						
29 or below	19	76.0	6	24.0	25	0.001
30 to 39	106	67.9	50	32.1	156	
40 to 49	352	69.8	152	30.2	504	
50 to 59	537	65.2	286	34.8	823	
60 to 69	673	66.3	342	33.7	1015	
70 to 79	440	61.2	279	38.8	719	
80 or above	50	50.0	50	50.0	100	
Sex						
Male	1430	63.6	820	36.4	2250	0.006
Female	747	68.4	345	31.6	1092	
Health Coverage Type						
National Health Insurance	2104	65.2	1125	34.8	3229	0.903
Medical aid	73	64.6	40	35.4	113	
Residential Area						
Rural	784	65.4	414	34.6	1198	0.784
City	1393	65.0	751	35.0	2144	
Premium Level						
Low	334	64.6	183	35.4	517	0.942
Middle	664	65.5	350	34.5	1014	
High	1179	65.1	632	34.9	1811	
Transfer (1st ct ≠ Surgery)						
No	2070	77.7	593	22.3	2663	<0.001
Yes	107	15.8	572	84.2	679	
Gastrectomy Type						
Total	400	54.5	334	45.5	734	<0.001
Subtotal/Partial	1777	68.1	831	31.9	2608	
Charlson Comorbidities Index						
	Mean	SD	Mean	SD		
Score	1.3	1.1	1.5	1.3		<0.001
Hospital Type, Taking the Initial ct						
Clinic	23	29.9	54	70.1	77	<0.001
Hospital	25	19.4	104	80.6	129	
General hospital	2129	67.9	1007	32.1	3136	
Hospital Provinces *, Taking the Initial ct						
Capital	940	71.9	367	28.1	1307	<0.001
Metropolitan	627	65.4	331	34.6	958	
Other rural provinces	610	56.6	467	43.4	1077	
Total	2177	65.1	1165	34.9	3342	

* Eighteen administrative districts in Korea are divided into three categories—capital, metropolitan, and provinces. The capital is Seoul, the six metropolitans are Busan, Daegu, Incheon, Daejeon, Gwangju, and Ulsan, and the rural provinces are the others.

**Table 2:** The adjusted odds ratios (aOR) for reexamination CT scans from March 2002 to December 2013.

Variable	OR	Low CI	High CI
Age Group			
29 or below	0.88	0.29	2.68
30 to 39	0.95	0.58	1.54
40 to 49	1.00		
50 to 59	1.24	0.93	1.66
60 to 69	1.31	0.98	1.74
70 to 79	1.56	1.15	2.12
80 or above	2.52	1.51	4.20
Sex			
Male	1.00		
Female	0.83	0.69	1.01
Health coverage type			
National health insurance	1.00		
Medical aid	0.93	0.55	1.55
Residential area			
Rural	1.00		
City	1.28	1.06	1.54
Premium level			
Low	1.00		
Middle	0.94	0.70	1.25
High	0.83	0.63	1.09
Transfer *			
No	1.00		
Yes	23.87	18.15	31.39
Gastrectomy type			
Total	1.90	1.55	2.33
Subtotal/Partial	1.00		
Charlson comorbidities index			
Per point	1.05	0.97	1.14
Hospital type, taking the initial ct			
Clinic	0.45	0.26	0.80
Hospital	1.40	0.79	2.46
General hospital	1.00		
Hospital provinces, taking the initial ct			
Capital (seoul)	1.00		
Metropolitan (busan, daegu, gwangju, daejeon, ulsan, and incheon)	0.45	0.26	0.80
Other rural provinces	1.40	0.79	2.46
Year			
Per one year	1.15	1.12	1.19

* Transfer means that the initial CT taking hospital is different from the surgery performed hospitals.

**Table 3:** The adjusted odds ratios (aORs) for reexamination CT scans among ‘transferred *’ patients.

Variable	OR	Low CI	High CI	*p*-Value
Transfer route				
General to general hospital	1.00			
Clinic and hospital to general hospital	0.65	0.32	1.33	0.2426
Number of beds, initial ct hospital				
Per 100 beds	0.88	0.83	0.94	<0.001
Number of beds, surgery hospital				
Per 100 beds	1.29	1.20	1.36	<0.001

* Age group, sex, health insurance, residential area, premium level, gastrectomy type, Charlson comorbidities index, hospital province taking the initial computer tomography scan, and year are adjusted. ‘Transfer’ means the transferring from any hospital (clinics, hospitals, and even other general hospitals) to general hospital level.

**Table 4:** The adjusted odds ratios (aOR) for reexamination CT scans among patients who took the initial CT at general hospitals.

VARIABLE	OR	Low CI	High CI	*p*-Value
Number of beds, initial ct hospital				
Per 100 beds	0.91	0.88	0.95	<0.001
Number of beds, surgery hospital				
Per 100 beds	1.10	1.06	1.13	<0.001

Age group, sex, health insurance, residential area, premium level, gastrectomy type, Charlson comorbidities index, hospital province taking the initial computer tomography scan, and year are adjusted.
